# Serum-derived exosomes from non-viremic animals previously exposed to the porcine respiratory and reproductive virus contain antigenic viral proteins

**DOI:** 10.1186/s13567-016-0345-x

**Published:** 2016-05-31

**Authors:** Sergio Montaner-Tarbes, Francesc E. Borrás, Maria Montoya, Lorenzo Fraile, Hernando A. del Portillo

**Affiliations:** Departamento de Producción Animal, ETSEA, Universidad de Lleida, Avenida Alcalde Rovira Roure 191, Lleida, Spain; Innovex Therapeutics SL, Badalona, Spain; IVECAT Group, Germans Trias i Pujol Health Science Research Institute (IGTP), Can Ruti Campus, 08916 Badalona, Spain; The Pirbright Institute, Ash Road, Pirbright, Surrey GU24 0NF UK; ICREA at ISGlobal, Barcelona Ctr. Int. Health Res. (CRESIB), Hospital Clínic-Universitat de Barcelona, 08036 Barcelona, Spain; ICREA at Institut d’Investigació Germans Trias i Pujol, Can Ruti Campus, 08916 Badalona, Spain

## Abstract

PRRSV is the etiological agent of one of the most important swine diseases with a significant economic burden worldwide and limitations in vaccinology. Exosomes are 30–100 nm vesicles of endocytic origin. Remarkably, immunizations with exosomes containing antigens from tumors or pathogens are capable of eliciting protective immune responses, albeit variably, in cancer and infectious diseases. Here we describe the isolation, molecular composition and immunogenicity of serum-derived exosomes from naïve animals, from PRRSV viremic animals and from animals previously PRRSV infected but already free of viruses (non viremic). Exosomes were isolated through size exclusion chromatography and characterized by different methodologies. Exosome-enriched fractions from naïve and natural infected animals contained classical tetraspanin exosomal markers (CD63 and CD81) and high concentrations of particles in the size-range of exosomes as detected by nanoparticle tracking analysis and cryo-TEM. NanoLC-MS/MS was used to identify viral antigens associated to exosomes. PRRSV-proteins were detected in serum samples from only viremic animals and from animals previously infected already free of viruses (non-viremic), but not in controls. Moreover, immune sera from pigs previously exposed to PRRSV specifically reacted against exosomes purified from non-viremic pig sera in a dose-dependent manner, a reactivity not detected when naïve sera was used in the assay. To facilitate future studies, a scaling-up process was implemented. To the best of our knowledge, this is the first molecular characterization of serum-derived exosomes from naïve pigs and pigs actively or previously infected with PRRSV. The presence of antigenic viral proteins in serum-derived exosomes free of virus, suggest their use as a novel vaccine approach against PRRSV.

**Electronic supplementary material:**

The online version of this article (doi:10.1186/s13567-016-0345-x) contains supplementary material, which is available to authorized users.

## Introduction

Porcine reproductive and respiratory virus (PRRSV) is the etiological agent of one of the most important swine diseases with a significant economic burden worldwide. Only in the US, it is estimated that $560 million yearly losses are directly related to this disease [1]. Current vaccines against PRRSV have focused on methods using modified live or attenuated virus [2], peptides [3], vectored vaccines [4], inactivated virus and subunit vaccines [5–7]. Available vaccines, however, have limitations such as little protective immunity [8], possible reversion to virulence [9], and incapability of eliciting long lasting and heterologous protection among European and American genotypes [10]. In addition, PRRSV strains have high antigenic variability and genetic polymorphisms [11, 12] and the highest mutation rate of RNA viruses [5]. All together, these limitations indicate that new alternatives to conventional vaccines are desperately needed aiming to control and eventually eradicating PRRSV.

Exosomes are 30–100 nm vesicles of endocytic origin originally described as a “garbage-disposable” mechanism of reticulocytes in their terminal differentiation to erythrocytes [13, 14]. This cellular origin and function were shown not to be unique as 10 years later, B-cells were also described to secrete exosomes with antigen presentation capacity and with the ability of generating specific T-cell responses [15]. Since these seminal observations, exosomes have been shown to be secreted by all immune cells and explored as novel vaccination approaches [16]. In fact, proof-of-principle Phase I clinical trials using dendritic cell-derived exosomes coupled to tumor-associated antigens have shown their safety and immunogenicity in cancer and Phase II trials are presently being conducted [17]. Of interest, antigens from infectious diseases associated with exosomes also demonstrated their capacity for eliciting specific and protective immune responses in preclinical mouse models [18–20]. For instance, vaccination with extracellular vesicles and exosomes can induce a strong immune response and increase survival in *Mycobacterium tuberculosis*, *Eimeria tenella*, *Toxoplasma gondii* [18, 19] and full protection against a lethal challenge in *Plasmodium yoelii* experimental infections [21]. Moreover, outer membrane vesicles (OMVs) derived from *Bordetella pertussis* used as vaccine in mice ameliorated infection following challenge with several strains [20]. For virus, exosomes play an important role not only involved in pathogenesis and virus spreading [22] but also in cell communication and protection against infection [23]. All together, these data strongly suggest the value of exosomes as a new vaccination approach in human health. Yet, no reports have shown their potential value for vaccination in animal health.

In this work, we describe the isolation and molecular composition of serum-derived exosomes obtained from naïve pigs, from viremic animals and from non-viremic animals previously infected with PRRSV. Our results unequivocally identified viral antigens associated to exosomes in viremic and non-viremic pigs. Moreover, viral proteins contained in serum-derived exosomes from non-viremic animals exhibit antigenic potential as judged by ELISA assays. A scaling-up protocol for obtaining serum-derived exosomes was also developed. Thus, opening the possibility of exploring these non-viremic nanovesicles as a novel vaccination approach against PRRSV.

## Materials and methods

### Samples

Sera were obtained from large white X Landrace pigs of approximately seventeen weeks of age that had suffered a PRRSV natural outbreak in two conventional farms and from animals of one PRRSV negative farm (naive pigs). The two PRRSV positive farms belong to the same integration company but from different sow origin. The PRRSV negative farm pertains to a different integration Company; thus, avoiding any confounding with samples. Viral as well as serological status of animals against PRRSV antigens were analyzed, respectively, by RT-PCR TaqMan® NA/EU PRRSV Reagents (Applied Biosystems) and IDEXX PRRS X3 Antibody Test (IDEXX). An independent diagnostics laboratory for porcine diseases in Lleida [24] confirmed these analyses following their own standard operational procedures.

Sera from all animals were classified as non-viremic (NV, PRRSV negative by RT-PCR) or viremic (V, PRRSV positive by RT-PCR), being both groups serologically positive to PRRSV using an IDEXX PRRS X3 Antibody Test. On the other hand, sera from naive control animals (CN) were PRRSV negative and free from antibodies against PRRSV. Details of sera used in this study are included in Additional file 1. All studies were approved by the ethical committee of the University of Lleida, Spain, and performed under their guidelines for animal care (DAAM7684).

### Exosome isolation: size-exclusion chromatography

Isolation of serum-derived exosomes by size exclusion chromatography (SEC) were performed as previously described [25]. Briefly, Sepharose CL-2B (Sigma-Aldrich, St. Louis, MO, USA) was packed in 10 mL syringes to a final volume of 10 mL and equilibrated with PBS-Citrate 0.32% (w/v). Frozen serum samples were thawed on ice, centrifuged at 500×*g* for 10 min at room temperature to remove cellular debris, and 2 mL aliquots were applied to each column. Collection of 20 fractions of 0.5 mL each started immediately using PBS-citrate as the elution buffer. Protein content of each fraction was analyzed using Bradford protein quantification assay according to manufacturer’s instructions (Bradford reagent, Sigma-Aldrich). To determine protein profiles, samples were loaded into 10% polyacrylamide BIORAD precast gels, separated at 120 V for 45 min and stained using SilverQuestTM Staining kit (Invitrogen).

### Flow cytometry analysis of molecular markers associated with extracellular vesicles

A bead-based assay for detection of two classical exosome markers, CD63 and CD81 was used to phenotypically identify SEC fractions containing exosomes [26]. Briefly, exosomes were coupled with Aldehyde/Sulfate Latex Beads, 4% w/v, 4 µm (Invitrogen) and then blocked with PBS 1X/BSA 0.1% (Sigma-Aldrich) /NaN3 0.01% (Sigma-Aldrich). Fractions were incubated in microtest conical bottom 96-well plates for 30 min at 4 °C with anti-CD63 and anti-CD81 antibodies (culture supernatant monoclonal antibodies) at 1:10 dilution. After washing, a 1/100 dilution of secondary antibody FITC (Southern Biotech) was incubated for 30 min at 4 °C. After removal of unbound secondary antibodies by centrifugation, beads were suspended in PBS and analyzed by flow cytometry using aBD FACSVerse (BD Biosciences) equipment. Median Fluorescence Intensity (MFI) and beads count data were obtained by FlowJo analysis Software of every sample-reading file.

### Nanoparticle tracking analysis (NTA)

Diameter size and concentration of vesicle population was determined using NanoSight LM10 equipment (Malvern). Fractions were evaluated using different dilutions in sterile-filtered PBS 1X (1/10 to 1/50) and the following parameters: camera at 30 frames per second (FPS), camera level at 16, temperature between 21–25 °C and video recording time 60 s in order to estimate the concentration and size distribution of vesicle population through light scattering and Brownian motion. Nanosight NTA Software analyzed raw data videos by triplicate and results were obtained in PDF datasheet with all selected values (Mean size, Median size, Mode size and concentration).

### Cryo-electron microscopy (Cryo-TEM)

Ten microliter aliquots from individual SEC fractions containing exosomes were directly laid on Formvar-Carbon EM grids and frozen in ethanol. Samples were analyzed on a Jeol JEM 2011 transmission electron microscope at an accelerating voltage of 200 kV. Samples and the 626 Gatan cryoholder were maintained at −182 °C during the whole process. To minimize electron bean radiation, images were recorded on a Gatan Ultrascan cooled CCD camera under low electron dose conditions. Vesicle size was determined using the ImageJ software (NIH) and setting calibration was performed pixels/nanometer.

### Mass spectrometry

Liquid Chromatography (nanoLCULTRA-EKSIGENT) followed by mass spectrometry (nanoLC-MS/MS) was performed on a LTQ Orbitrap Velos (Thermo Fisher). Briefly, samples were reduced with 10 mM DTT (Dithiothreitol), alkylated with 55 mM iodoacetamide and precipitated by 10% TCA. After washing with acetone, 2 μL of 8 M urea were added and samples brought to a final concentration of 1.6 M urea. One microgram of trypsin (*Sus scrofa*) was added and digestions were carried overnight at 37 °C. The reaction was stopped with 1% formic acid. The amount of sample submitted to mass spectrometry analyses was based on nanoparticle tracking analysis (see below) and ranged from 9.8 × 10^7^ to 3.9 × 10^8^ particles/mL among all samples analyzed. MS/MS was performed in the LTQ using data dependent dynamic exclusion of the top 20 most intense peptides using repeat count = 1, repeat duration = 30 s, exclusion list size of 500 and exclusion list duration = 30 s as a parameters. The top 20 most intense peptides were isolated and fragmented by low energy CID, 35% collision energy.

### Database search and protein identification

Raw spectral data from Xcalibur™ (Thermo Scientific, v2.1) was searched against a custom database compiled from [27] in FASTA format for uploading it into Andromeda Search Engine 1.4. The database contained complete and partial sequences of PRRSV (22 976 sequences) and *Sus scrofa* (59 898 sequences). The sequence for trypsin from *Sus scrofa* (Accession P00761 from [28]) and default contaminant database were also included in the search carried out with Maxquant 1.5/Andromeda 1.4 software. Contaminants and proteins identified only by site modification were filtered out from the list. Proteins found in all groups were scored positive if they had at least two unique peptides and 1% False Discovery Rate (FDR) for protein and peptide identification. After filtering, proteins of each group were compared in a Venn diagram using Venny 2.0 software [29] to determine which proteins were unique and shared among samples.

### Gene ontology (GO) analyses by PANTHER overrepresentation test

Porcine proteins identified by Maxquant 1.5/Andromeda 1.4 Software were filtered by elimination of “contaminants” and “Only identified by site proteins”. Then, the most common proteins with highest score were summited to UniprotKb “retrieve/ ID mapping [28] to convert GI numbers (Maxquant results) to UniprotKB ID number and eliminating redundant hits. Then, the final protein list (184) (.tab format) was summited to PANTHER Overrepresentation Test (release 20150430) [30] using Annotation Version: PANTHER version 10.0 Released 2015-05-15, Reference List: *Sus scrofa* (all genes in database) and perform all three GO-Slim analysis available (Biological process, Cellular component and Molecular function) [31]. Also, exosomal proteins of *Sus scrofa* were compared against exosome proteins of different farm animals (*Bos taurus*, *Equus caballus*, *Gallus gallus* and *Rattus norvegicus*) using Funrich analysis software [32].

### ELISA assays

An indirect ELISA protocol was initially performed (dilution chessboard) for titration of sera coming from NV, V and CN pigs using a secondary antibody Goat anti-Pig IgG (Fc): HRP (AbSerotec AAI41P) and Porcillis PRRSV vaccine as coating antigen (Intervet Lot. A200ED03) (Additional file 2). Using a range of sera dilutions previously titrated, circulating IgG antibodies from NV and CN pigs were tested in a double ELISA test against homologous NV serum-derived exosomes (sandwich ELISA) and against whole viral vaccine (Porcillis PRRS Vaccine “intervet” lot. A200ED03) as previously described. For sandwich ELISAs, plates were first coated with anti-CD63 antibodies and after washing and blocking, SEC fractions (100 uL per well) containing exosomes were incubated 90 min at 37 °C. Sera samples were afterwards incubated for 1 h at room temperature, washed and incubated with secondary antibody Goat anti-Pig IgG (Fc): HRP (AbSerotec AAI41P) at 1:10 000 dilution and optical density was measured at 450 nm using Varioskan equipment (Thermo Scientific).

### Scaling-up process for vesicle enrichment and isolation

The process of polyethylene-glycol (PEG) precipitation was based on scale-up process for retrovirus stock in order to maintain structure and functionality of extracellular vesicles [33, 34]. Thus, two adult healthy animals (80–100 kg) were anesthetized and approximately 500 mLs of peripheral blood from each animal collected by venous puncture. Blood was collected into 50 mL Falcon tubes to facilitate collection of sera and minimize hemolysis. Sera were precipitated overnight at room temperature by adding PEG at 8.5% w/v ratio. Pellets were resuspended in 20 mLs of PBS and loaded into PuriFlash Dry Load Columns 80G (Interchim) filled with 100 mL of sepharose CL-2B (separation matrix) and 5 mLs fractions collected for further analysis.

## Results

### Characterization of serum-derived exosomes after purification by size exclusion chromatography

**Figure 1:**
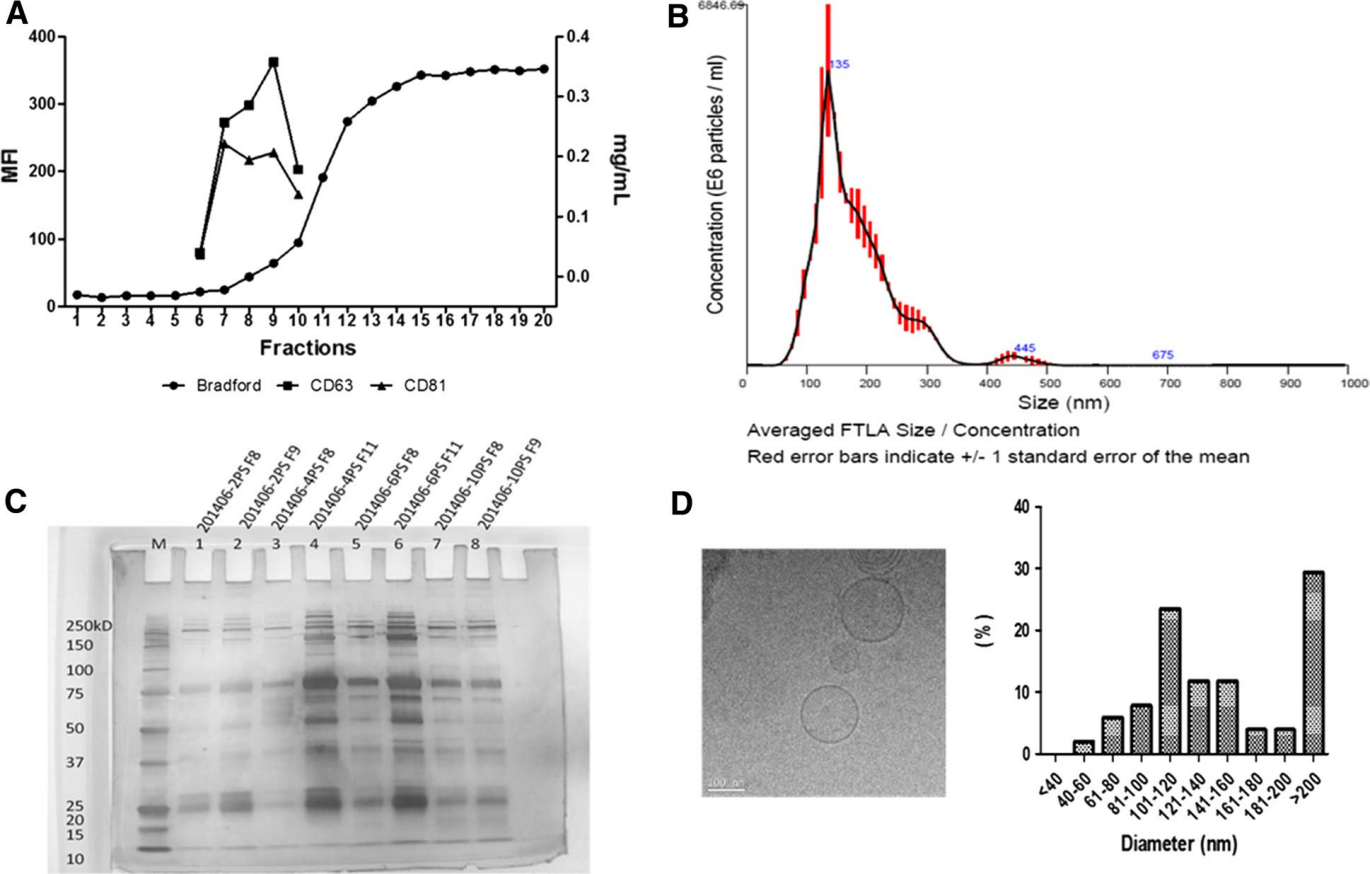
**Characterization of porcine serum-derived exosomes sera by different methodologies.** Bradford and flow cytometry analyses (**A**), nanoparticle tracking analysis (**B**), cryo-electron microscopy (**C**) and SDS-PAGE/Silver Staining (**D**) are represented. MFI: Median fluorescence unit, mg/mL: Bradford measure unit, M: Molecular weight marker, F6-F9: Fraction number from SEC and percentage (%) size distribution (nm).

SEC was used to isolate exosomes from sera of naïve animals (CN) pertaining to a PRRSV negative farm, and sera from viremic animals (V) or non-viremic (NV) animals from two PRRSV positive farms where different PRRS viruses were detected. Preliminary studies on sequence polymorphisms from PRRSV viruses isolated in these farms revealed 85% homology among them (data not shown). Twenty 0.5 mL aliquot fractions were collected from each serum and individually analysed for (i) for their protein content and (ii) for the presence of two “classical” exosome markers, CD63 and CD81. In all samples analysed, exosome markers were identified in fractions 7–10, whereas total protein content increased in later fractions (Figure 1A). In addition, NTA revealed that preparations from all animals were highly homogeneous in terms of particle size (100–200 nm with a medium size of 127 nm) and concentration (10^9^–10^10^ particles per mL) (Figure 1B; Additional file 3). Furthermore, electrophoresis of the protein content of SEC fractions from different samples revealed a similar profile in early fractions and, as expected, an enrichment of plasma proteins in late fractions (Figure 1C). Vesicle size and structure were also analysed by cryo-TEM. Similar to NTA, vesicles from 80–200 nm in diameter were predominantly observed whereas electro-dense bodies typically associated with viral particles, were not (Figure 1D).

### Proteomic analysis

**Figure 2:**
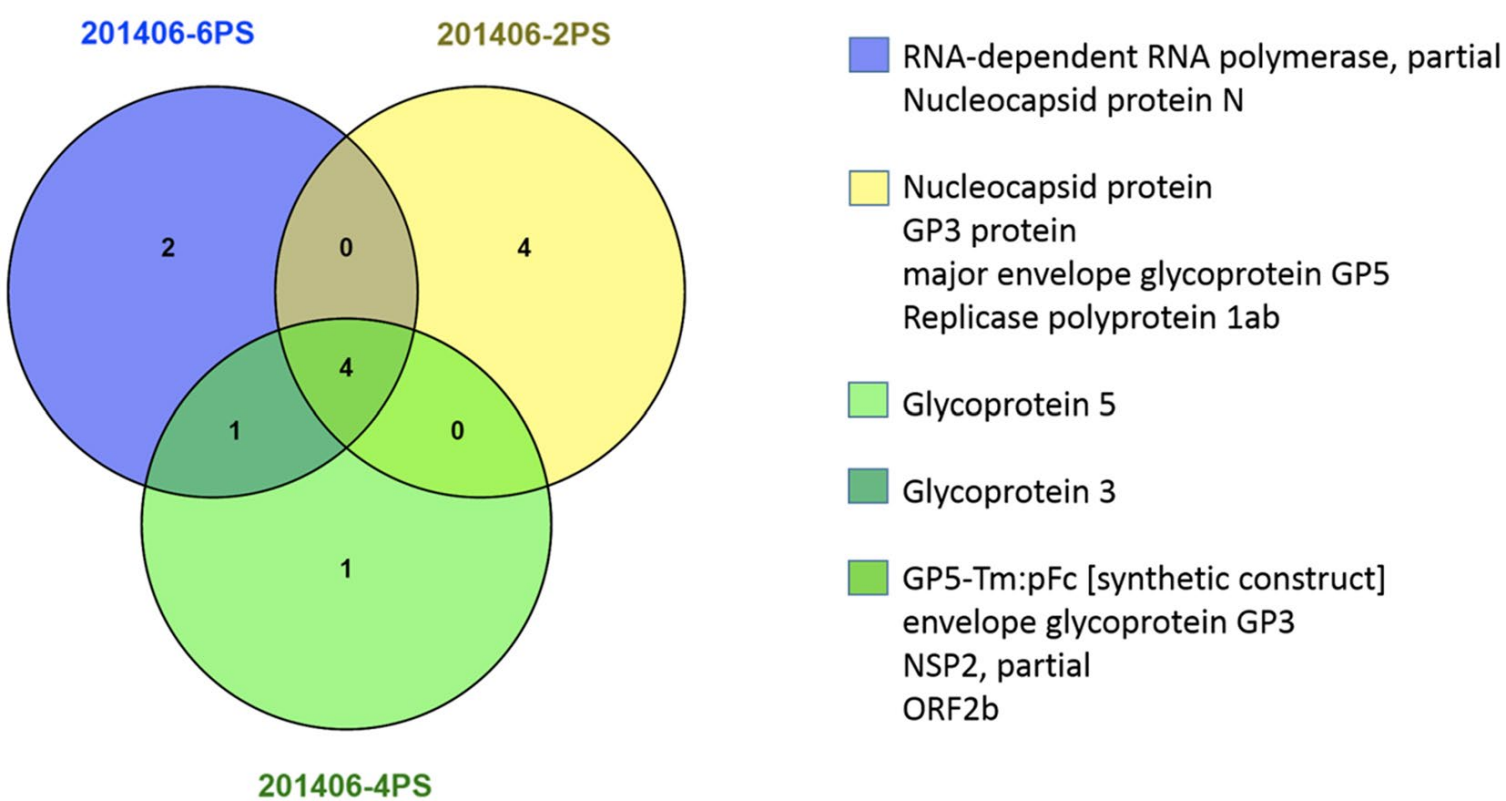
**Venn diagram showing overlap and unique peptides of viral proteins detected in different sample groups: non-viremic (201406-6PS) and viremic (201406-2PS and 201406-4PS).** The overlapping and unique peptides identified for proteins in these sample groups are shown.

To characterize the exosome protein composition from different groups of animals, liquid chromatography and mass spectrometry were applied for protein sequencing and identification from samples of one NV animal and two V animals (Figure 2). Of importance, peptides pertaining to viral proteins were identified in serum-derived exosomes from all animals whereas others were uniquely identified. Thus, peptides from major envelope glycoprotein GP5-Tm:pFc (a fusion protein of GP5 with no transmembrane domain and pig fragment crystallizable portion), from envelope glycoprotein GP3, NSP2 and partial ORF2b were detected in exosomes from all (NV and V) animals. Other peptides from nucleocapsid protein, envelope glycoprotein GP3 protein, major envelope glycoprotein GP5 and replicase polyprotein 1ab, where only identified in exosomes from V animals whereas peptides from envelope glycoprotein 3 were identified in exosomes from one V and one NV animal. Interestingly, peptides from RNA-dependent RNA polymerase and nucleocapsid protein N, were detected only in exosomes from NV animals.

To confirm the presence of unique viral proteins in exosomes from NV animals, three additional samples were also analysed by liquid chromatography and mass spectrometry and confirmed the presence of RNA-dependent RNA polymerase, partial and nucleocapsid protein N. None of these proteins were found in serum-derived exosomes from naïve animals (CN) further confirming the specificity of these results (not shown).

The proteomics analysis also identified more than 400 porcine proteins contained within exosomes (Additional file 4). Of interest, GO analysis showed an important enrichment of lipid transport, response to external stimulus, proteolysis, enzymatic activities and extracellular space proteins, all related to exosomes composition and function (Table 1). Besides, when comparing exosome porcine proteins in our database with exosomes from other farm animals using the Funrich software [32], 48 proteins were shared among *B. taurus* and *S. scrofa* (approximately 2.9% of total proteins), 5 with *E. caballus*, 6 with *G. gallus* and 3 with *R. norvegicus* as outlier in the evolutionary line (Additional file 5). Interestingly, even though the pig protein database is smaller than others in these analyses, there is a coincidence of 3% with *Bos taurus*, and at least 4 of these proteins are classical exosomal markers (CD5, CD9, CD81 and CD63) (Additional file 5).

**Table 1:** **Gene Onthology analysis of** ***Sus scrofa*** **proteins detected in exosomal enriched fractions**

	Ref list. 21483	Exp. list (91)	Expected	Over/under	Fold enrichment	*P* value
PANTHER GO-slim biological process						
Lipid transport	305	10	1.29	+	>5	1.67E−04
Response to external stimulus	347	10	1.47	+	>5	5.24E−04
Proteolysis	690	12	2.92	+	4.11	7.98E−03
Response to stress	659	11	2.79	+	3.94	2.48E−02
Immune system process	1399	17	5.93	+	2.87	1.65E−02
Localization	2610	28	11.06	+	2.53	4.69E−04
Transport	2484	26	10.52	+	2.47	1.93E−03
Response to stimulus	2364	23	10.01	+	2.3	2.31E−02
Unclassified	9376	24	39.72	−	0.6	0.00E+00
PANTHER GO-slim molecular function						
Lipid transporter activity	106	8	0.45	+	>5	3.51E−06
Peptidase inhibitor activity	227	10	0.96	+	>5	8.65E−06
Serine-type peptidase activity	293	11	1.24	+	>5	8.91E−06
Enzyme inhibitor activity	337	10	1.43	+	>5	3.08E−04
Peptidase activity	605	16	2.56	+	>5	9.26E−07
Receptor binding	947	18	4.01	+	4.49	1.42E−05
Hydrolase activity	2181	23	9.24	+	2.49	5.06E−03
Protein binding	2729	28	11.56	+	2.42	8.53E−04
Unclassified	10 794	35	45.72	−	0.77	0.00E+00
PANTHER GO-slim cellular component						
Extracellular space	6	3	0.03	+	>5	1.22E−04
Extracellular region	624	18	2.64	+	>5	6.31E−09
Unclassified	17 295	62	73.26	−	0.85	0.00E+00

### Specific immune recognition of PRRSV-proteins in serum-derived exosomes

**Figure 3:**
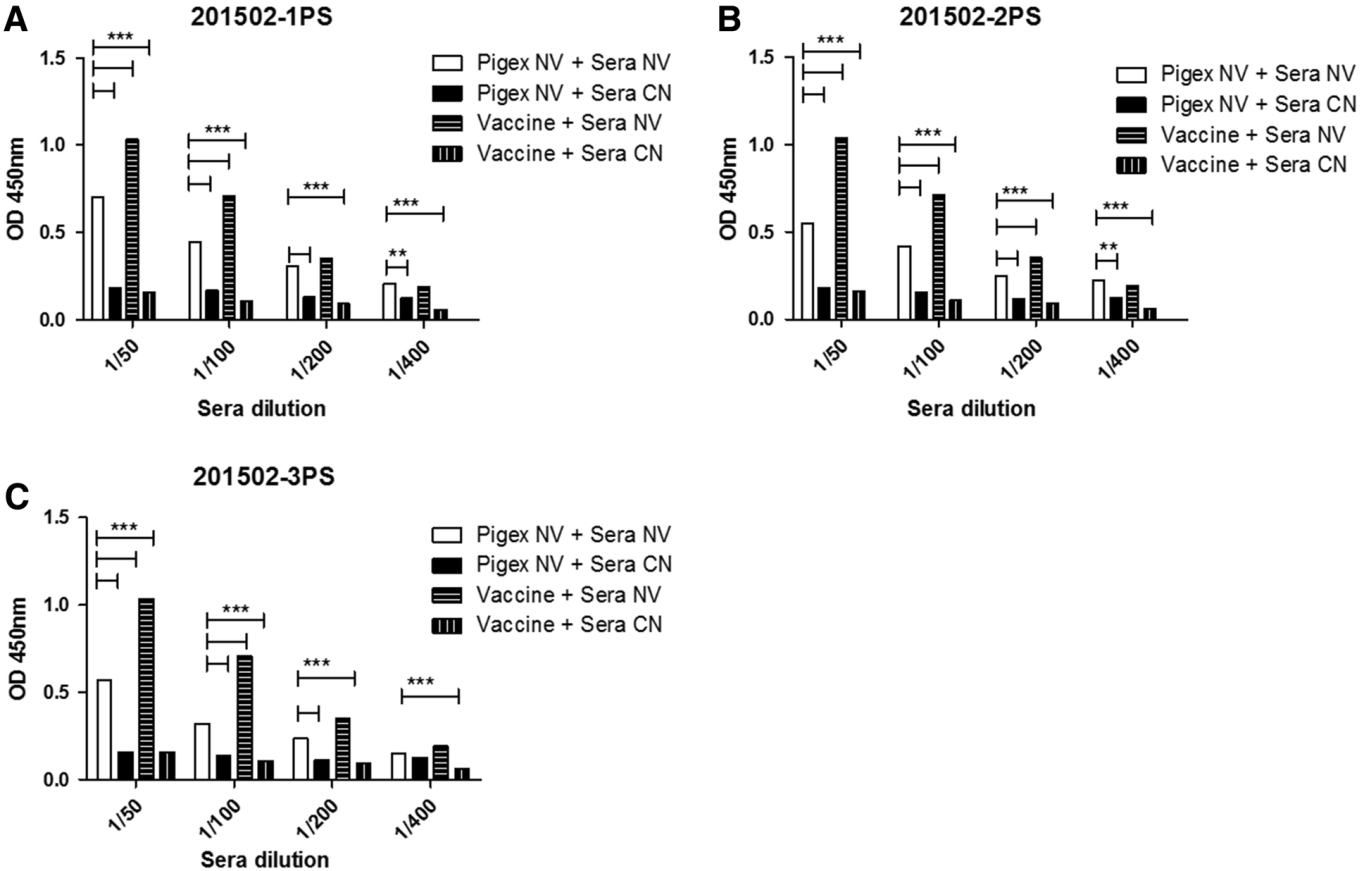
**ELISA assay for evaluation of NV and CN porcine sera immune recognition over inactivated viral particle PRRSV vaccine (Porcilis PRRS, Intervet) and exosomes derived from NV porcine sera of different origin.** Analyses of naïve (CN) and non-viremic (NV) sera against exosomes derived from sample 201502-1PS (**A**), 201502-2PS (**B**) and 201502-3PS (**C**). Optical density (OD) was measured at 450 nm and it is represented in the “Y” axis. “X” axis shows the dilution factor for sera samples (1/50 to 1/400). For each animal, exosomes were isolated and captured using anti-CD63 antibody and tested against both sera (**p < 0.01; ***p < 0.0001).

**Figure 4:**
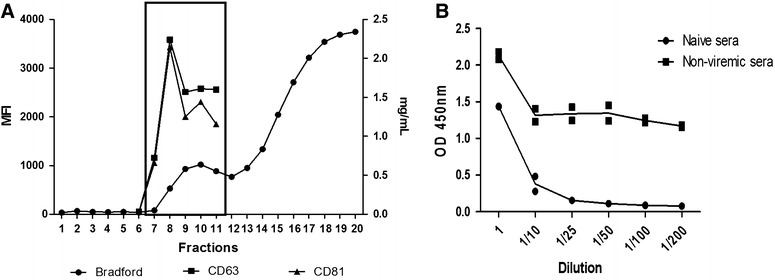
**Characterization of exosomes isolated using PEG/SEC methodology from porcine sera.** Bradford and flow cytometry analyses (**A**), Sandwich ELISA for exosomes derived from non-viremic animal (**B**), are represented. MFI: Median fluorescence unit, mg/mL: Bradford measure unit, CD63-CD81 are molecular markers for exosomes characterization. For FACS and Bradford analyses is evident that higher values are represented in comparison with non-concentrated samples (10-fold change). In addition, the indirect quantification through Bradford of protein associated with exosomes it is possible when sera is concentrated through PEG and separated using SEC, making this an important tool for further evaluation of immunogenic properties in vitro and in vivo. For the sandwich ELISA, exosomes derived from non-infected animal were tested against two types of porcine sera. Optical density (OD) was measured at 450 nm and represented in the “Y” axis. “X” axis shows the dilution factor for exosomes samples (1 to 1/200). Duplicated values are represented as squares and dots and mean as a line.

To determine whether serum-derived exosomes from NV animals contained antigenic viral proteins, swine sera was tested by indirect and sandwich ELISA. First, sera was titrated using Porcilis PRRSV vaccine as coating antigen and showed maximum differences between CN and NV animals at 1/50–1/100 dilutions and 1/10 000 dilution of the secondary antibody without being at saturation values (Additional file 2). As shown in Figure 3, statistically significant differences (*p* < 0.05) between sera from NV and CN animals using three individual NV exosome preparations (1PS, 2PS and 3PS) at 1/50 and 1/100 dilutions, were observed. Moreover, similar reactivity and statistical significance were observed when sera from CN and NV animals were tested against a commercially available vaccine (Porcillis PRRSV vaccine, Intervet) as the coating antigen. In addition, evaluation of antigenicity was done in concentrated exosome samples obtained through PEG/SEC isolation. Non-viremic sera but not naïve was able to recognize exosomes derived from non-viremic animals in a dose dependent manner (Figure 4).

### Scaling-up process for vesicle enrichment and isolation

Approximately half litre of peripheral blood was obtained from each of two anesthetized animals and their sera collected following standard procedures. Sera were precipitated using PEG, pellets suspended into 20 mL aliquots and passed through individual Sepharose CL-2B 100 mL columns. Results demonstrated that this escalation procedure yielded purified exosomes with the same NTA profile, SDS-PAGE and cryo-EM as those obtained from 2 mL aliquots of serum (Figure 4). Noticeably, the yield was significantly increased as proteins were detected by the Bradford assay in SEC fractions containing exosomes (Figure 4A) and NTA analysis revealed a twofold increase in the number of particles (10^10–11^ particles/mL) as opposed to those obtained from 2 mLs (10^8–9^ particles/mL). Last, PEG-precipitation did not affect the immunological properties of exosomes as immune sera similarly and specifically recognized them (Figure 4B).

## Discussion

Here, for what we believe is the first time, we describe the isolation, characterization, antigenicity and scaling-up process of serum-derived exosomes from naïve pigs and from pigs actively or previously infected with PRRSV.

Firstly, size-exclusion chromatography was applied to analyze serum-derived exosomes from pigs in a small and scaling-up procedure. This single-standing methodology is presently considered a solid and reproducible method for isolation and characterization of extracellular vesicles in the size range of exosomes from different biological fluids such as plasma [25, 35], saliva [36] and urine [37]. In fact, it removes most contaminant abundant proteins and purifies 100–200 nm particles associated with classical exosomal markers. Accordingly, our results showed that exosomes eluted in fractions 7 to 10, whereas more abundant serum proteins (such as albumin), as judged by the Bradford assay, eluted in later fractions. Moreover, two “classical” exosomal markers, CD63 and CD81 [38] showed maximum MFI values in these same fractions (Figures 1, 4), where high concentrations of particles in the size-range of exosomes were also detected by NTA and cryo-TEM analyses and low protein content was detected. Of interest, bicosome-like structures (vesicles within vesicles) were observed in agreement with similar structures found in structural studies from other fluids [39–41]. These results strongly suggest that porcine serum samples have similar and reproducible SEC elution profiles as described in human samples, reinforcing the use of SEC as a single-standing and easily implementing technology facilitating field studies of extracellular vesicles in animal diseases of veterinary importance.

To identify PRRSV viral proteins associated with serum-derived exosomes, we used nanoLC-MS/MS. Remarkably, RNA-dependent RNA polymerase, partial and nucleocapsid protein N, were detected only in exosomes from the non-viremic animals (Figure 2). The nucleocapsid protein (N) is one of the most abundant and immunodominant viral proteins during PRRS infection [42]. This protein interacts with different cellular factors of the host to facilitate virus infection and its role is crucial for mature viral particle formation within the cell, binding to the viral RNA genome and replication machinery including RNA-dependent RNA polymerase, and also interacts with itself to form the core capsid [43, 44]. Of interest, the N protein and three non-structural (Nsps) PRRSV proteins have been identified as playing an important role in type I IFN suppression and modulation of the NF-kB pathway as it is translocated to the nucleus during early stages of infection [43, 45]. Late in infections, nucleocytoplasmic transport of the N protein increase the cytoplasmic concentration of this protein. It is tempting to speculate that an increase in virus N protein at cytoplasm during chronic infections might favor the release of the N-protein and RNA-dependent RNA polymerase in exosomes, which could at least partially explain the data from the proteomic analysis. In the absence of supporting data, this remains to be further demonstrated. Regardless, N proteins has been expressed in different models such as *Baculovirus* and *Escherichia coli* [42] and soya been seeds [46]; interestingly, in all cases it was capable of inducing both cellular and humoral immune response (murine model) or being recognized by PRRSV immune porcine sera.

To test the antigenic properties of serum-derived exosomes from previously infected animals, we first captured exosomes isolated from three non-viremic (NV) animals that were free of detectable virus (RT-PCR negative) at the time of sera collection. Analysis of serum-derived exosomes from V animals was not performed in sandwich ELISA as PRRSV virus has the same density and size of exosomes; thus, confounding such analyses. Immune sera from pigs previously exposed to PRRSV, specifically reacted to these exosomes in a dose-dependent manner and similar, albeit at lower values, to antigens contained in the Porcillis attenuated vaccine; these results thus demonstrate that viral proteins contained in the exosome preparation from NV animals are antigenic. The immunogenic properties of exosomes containing pathogen-associated antigens have been tested in several preclinical models and diseases [18, 19, 47]. Yet, to the best of our knowledge, no reports are presently available on antigenic properties of serum-derived exosomes with no pathogen load detected in peripheral circulation. This observation may be of importance for future vaccine approaches.

As a *bonafide* aspect of these analyses, we present the first proteomics analysis of pig proteins contained in serum-derived exosomes (Additional file 2). More than 400 porcine proteins associated with lipid transport, response to external stimulus, response to stress, immune system processes, some enzymes and extracellular space proteins are enriched in our exosomal fractions indicating cargo-selection related to cell communication and metabolic processes. These proteins thus represent a first baseline proteome of porcine serum-derived exosomes facilitating future studies between host and pathogens in PRRSV and other animal diseases.

The use of nanovesicles in vaccine approaches against PRRSV is bringing new and recent exciting data. It has been previously reported that nanoparticle entrapped antigens are more effective than conventional vaccine platforms [48–50] and demonstrated increasing titers of virus neutralizing antibodies in serum and lungs. Additionally, a different kind of artificial exosome was used to deliver microRNA into porcine alveolar macrophages (PAMs) to suppress expression of CD163 or Sialoadhesin receptors in cell surface making those less susceptible to PRRSV infection and replication [51]. Our results, including a scaling-up process, thus warrant further exploring serum-derived exosomes from PRRSV infections as a different vaccination approach. Regulatory aspects, similar to what has been recently positioned by the International Society of Extracellular Vesicles on human health [52], should not encounter major obstacles in future animal trials.

## Additional files

10.1186/s13567-016-0345-x **Codification and status of virus in blood and antibodies in porcine sera samples received.** All samples were codified as soon as received in the laboratory and classified by virus and antibody status according to the RT-PCR and serology test for PRRSV.
10.1186/s13567-016-0345-x **ELISA chessboard plate assay for standardization of NV, CN and V porcine sera immune recognition over inactivated viral particle PRRSV vaccine (Porcilis PRRS, Intervet).** Analyses of different dilutions of secondary antibody (A) 1/100, (B) 1/1000 (C) 1/10 000 and (D) 1/10 000 for each type of sera. Optical density (OD) was measured at 450nm and it is represented in the “Y” axis. “X” axis shows the dilution factor for sera samples (1/5 to 1/5000) and each bar represents a dilution for secondary antibody (1/100 to 1/100 000). The dilution used for further experiments was selected by comparison of differences between non-viremic and naïve samples and selecting the ones showing highest difference combined with highest dilution without signal saturation (1/50 for sera and 1/10 000 for secondary antibody).
10.1186/s13567-016-0345-x **Naïve and viremic protein, FACS and nanosight profile. (A) (C) Viremic sample 201406-2PS (B) (D) Naïve sample 201406-CN2.** Viremic and naïve samples of sera were evaluated by the same methodology as non-viremic samples to detect any differences between groups. Protein and molecular markers profile were similar as already seen with non-viremic samples, where fractions enriched with exosomes exhibit higher MFI values for CD63 and CD81 even though there is no measurable protein detected by Bradford assay.
10.1186/s13567-016-0345-x ***Sus scrofa*** **proteins identified by Maxquant/Andromeda analysis using nanoLC-MS/MS spectra.** Porcine proteins were identified in exosomes by proteomic analyses and several molecular markers of exosomes are present in these samples such as CD5, CD9, CD81 and CD63.
10.1186/s13567-016-0345-x **Venn diagram of farm animal exosomal proteins and coincidence table comparing** ***Sus scrofa*** **and** ***Bos taurus***. Exosome proteins from different farm animal species obtained from Vesiclepedia were used to compare with our Sus scrofa exosomal proteins. Similarities among species in exosomal proteins are represented in the venn diagram.

## References

[1] Neumann EJ, Kliebenstein JB, Johnson CD, Mabry JW, Bush EJ, Seitzinger AH, Green AL, Zimmerman JJ (2005). Assessment of the economic impact of porcine reproductive and respiratory syndrome on swine production in the United States. J Am Vet Med Assoc.

[2] Charerntantanakul W (2012). Porcine reproductive and respiratory syndrome virus vaccines: Immunogenicity, efficacy and safety aspects. World J Virol.

[3] Wang X, Qui L, Dang Y, Xiao S, Zhang S, Yang Z (2014). Linear epitope recognition antibodies strongly respond to the C-terminal domain of HP-PRRSV GP5. Vet Microbiol.

[4] Cruz JLG, Zúñiga S, Bécares M, Sola I, Ceriani JE, Juanola S, Plana J, Enjuanes L (2010). Vectored vaccines to protect against PRRSV. Virus Res.

[5] Renukaradhya GJ, Meng X-J, Calvert JG, Roof M, Lager KM (2015). Inactivated and subunit vaccines against porcine reproductive and respiratory syndrome: current status and future direction. Vaccine.

[6] Renukaradhya GJ, Meng X-J, Calvert JG, Roof M, Lager KM (2015). Live porcine reproductive and respiratory syndrome virus vaccines: current status and future direction. Vaccine.

[7] Renukaradhya GJ, Dwivedi V, Manickam C, Binjawadagi B, Benfield D (2012). Mucosal vaccines to prevent porcine reproductive and respiratory syndrome: a new perspective. Anim Heal Res Rev.

[8] Pileri E, Gibert E, Soldevila F, García-Saenz A, Pujols J, Diaz I, Darwich L, Casal J, Martín M, Mateu E (2015). Vaccination with a genotype 1 modified live vaccine against porcine reproductive and respiratory syndrome virus significantly reduces viremia, viral shedding and transmission of the virus in a quasi-natural experimental model. Vet Microbiol.

[9] Martínez-Lobo F, de Lome L, Díez-Fuertes F, Segalés J, García-Artiga C, Simarro I, Castro J, Prieto C (2013). Safety of porcine reproductive and respiratory syndrome modified live virus (MLV) vaccine strains in a young pig infection model. Vet Res.

[10] Han K, Seo HW, Park C, Chae C (2014). Vaccination of sows against type 2 Porcine Reproductive and Respiratory Syndrome Virus (PRRSV) before artificial insemination protects against type 2 PRRSV challenge but does not protect against type 1 PRRSV challenge in late gestation. Vet Res.

[11] Music N, Gagnon CA (2010). The role of porcine reproductive and respiratory syndrome (PRRS) virus structural and non-structural proteins in virus pathogenesis. Anim Heal Res Rev.

[12] Kappes MA, Faaberg KS (2015). PRRSV structure, replication and recombination: origin of phenotype and genotype diversity. Virology.

[13] Pan BT, Johnstone RM (1983). Fate of the transferrin receptor during maturation of sheep reticulocytes in vitro: selective externalization of the receptor. Cell.

[14] Harding C, Heuser J, Stahl P (1983). Receptor-mediated endocytosis of transferrin and recycling of the transferrin receptor in rat reticulocytes. J Cell Biol.

[15] Raposo G, Nijman HW, Stoorvogel W, Liejendekker R, Harding CV, Melief CJ, Geuze HJ (1996). B lymphocytes secrete antigen-presenting vesicles. J Exp Med.

[16] Théry C (2011). Exosomes: secreted vesicles and intercellular communications. F1000 Biol Rep.

[17] Chaput N, Théry C (2011). Exosomes: immune properties and potential clinical implementations. Semin Immunopathol.

[18] Schorey JS, Bhatnagar S (2008). Exosome function: from tumor immunology to pathogen biology. Traffic.

[19] Marcilla A, Martin-Jaular L, Trelis M, de Menezes-Neto A, Osuna A, Bernal D, Fernandez-Becerra C, Almeida IC, Del Portillo HA (2014). Extracellular vesicles in parasitic diseases. J Extracell Vesicles.

[20] György B, Hung ME, Breakefield XO, Leonard JN (2015). Therapeutic applications of extracellular vesicles: clinical promise and open questions. Annu Rev Pharmacol Toxicol.

[21] Martin-Jaular L, Nakayasu ES, Ferrer M, Almeida IC, Del Portillo HA (2011). Exosomes from Plasmodium yoelii-infected reticulocytes protect mice from lethal infections. PLoS One.

[22] Izquierdo-Useros N, Naranjo-Gómez M, Erkizia I, Puertas MC, Borràs FE, Blanco J, Martinez-Picado J (2010). HIV and mature dendritic cells: Trojan exosomes riding the Trojan horse?. PLoS Pathog.

[23] Bonito P, Ridolfi B, Columba-Cabezas S, Giovannelli A, Chiozzini C, Manfredi F, Anticoli S, Arenaccio C, Federico M (2015). HPV-E7 Delivered by engineered exosomes elicits a protective CD8 + T cell-mediated immune response. Viruses.

[24] Grup de Sanejament Porcí. http://www.gsplleida.net/. Accessed 19 Nov 2015.

[25] Böing AN, van der Pol E, Grootemaat AE, Coumans FAW, Sturk A, Nieuwland R (2014). Single-step isolation of extracellular vesicles by size-exclusion chromatography. J Extracell Vesicles.

[26] Théry C, Amigorena S, Raposo G, Clayton A (2006). Isolation and characterization of exosomes from cell culture supernatants and biological fluids. Curr Protoc Cell Biol.

[27] NCBI protein Database. http://www.ncbi.nlm.nih.gov/protein. Accessed 15 Dec 2015.

[28] UniProt Knowledgebase. http://www.uniprot.org/uniprot/. Accessed 12 Jan 2016.

[29] Venny. An interactive tool for comparing lists with Venn’s diagrams. http://bioinfogp.cnb.csic.es/tools/venny/index.html. Accessed 15 Dec 2015.

[30] Protein ANalysis THrough Evolutionary Relationships. http://pantherdb.org/. Accessed 24 Feb 2016.

[31] Mi H, Muruganujan A, Casagrande JT, Thomas PD (2013). Large-scale gene function analysis with the PANTHER classification system. Nat Protoc.

[32] Pathan M, Keerthikumar S, Ang C-S, Gangoda L, Quek CYJ, Williamson NA, Mouradov D, Sieber OM, Simpson RJ, Salim A, Bacic A, Hill AF, Stroud DA, Ryan MT, Agbinya JI, Mariadason JM, Burgess AW, Mathivanan S (2015). FunRich: an open access standalone functional enrichment and interaction network analysis tool. Proteomics.

[33] Cepko C (2001). Large-scale preparation and concentration of retrovirus stocks. Curr Protoc Mol Biol.

[34] Kordelas L, Rebmann V, Ludwig A-K, Radtke S, Ruesing J, Doeppner TR, Epple M, Horn PA, Beelen DW, Giebel B (2014). MSC-derived exosomes: a novel tool to treat therapy-refractory graft-versus-host disease. Leukemia.

[35] de Menezes-Neto A, Sáez MJF, Lozano-Ramos I, Segui-Barber J, Martin-Jaular L, Estanyol Ullate JM, Fernandez-Becerra C, Borrás FE, del Portillo HA (2015). Size-exclusion chromatography as a stand-alone methodology identifies novel markers in mass spectrometry analyses of plasma-derived vesicles from healthy individuals. J Extracell Vesicles.

[36] Ogawa Y, Kanai-Azuma M, Akimoto Y, Kawakami H, Yanoshita R (2008). Exosome-like vesicles with dipeptidyl peptidase IV in human saliva. Biol Pharm Bull.

[37] Lozano-Ramos I, Bancu I, Oliveira-Tercero A, Pilar Armengol M, Menezes-Neto A, Del Portillo HA, Lauzurica-Valdemoros R, Borràs FE (2015). Size-exclusion chromatography-based enrichment of extracellular vesicles from urine samples. J Extracell Vesicles.

[38] Kim D-K, Lee J, Kim SR, Choi D-S, Yoon YJ, Kim JH, Go G, Nhung D, Hong K, Jang SC, Kim S-H, Park K-S, Kim OY, Park HT, Seo JH, Aikawa E, Baj-Krzyworzeka M, van Balkom BWM, Belting M, Blanc L, Bond V, Bongiovanni A, Borràs FE, Buée L, Buzás EI, Cheng L, Clayton A, Cocucci E, Dela Cruz CS, Desiderio DM (2015). EVpedia: a community web portal for extracellular vesicles research. Bioinformatics.

[39] Yuana Y, Koning RI, Kuil ME, Rensen PCN, Koster AJ, Bertina RM, Osanto S (2013). Cryo-electron microscopy of extracellular vesicles in fresh plasma. J Extracell Vesicles.

[40] Issman L, Brenner B, Talmon Y, Aharon A (2013). Cryogenic transmission electron microscopy nanostructural study of shed microparticles. PLoS One.

[41] Höög JL, Lötvall J (2015). Diversity of extracellular vesicles in human ejaculates revealed by cryo-electron microscopy. J Extracell Vesicles.

[42] Rodriguez MJ, Sarraseca J, Garcia J, Sanz A, Plana-Duran J, Casal JI (1997). Epitope mapping of the nucleocapsid protein of European and North American isolates of porcine reproductive and respiratory syndrome virus. J Gen Virol.

[43] Sun Y, Han M, Kim C, Calvert JG, Yoo D (2012). Interplay between interferon-mediated innate immunity and porcine reproductive and respiratory syndrome virus. Viruses.

[44] Liu L, Lear Z, Hughes DJ, Wu W, Zhou E, Whitehouse A, Chen H, Hiscox JA (2015). Resolution of the cellular proteome of the nucleocapsid protein from a highly pathogenic isolate of porcine reproductive and respiratory syndrome virus identifies PARP-1 as a cellular target whose interaction is critical for virus biology. Vet Microbiol.

[45] Wulan WN, Heydet D, Walker EJ, Gahan ME, Ghildyal R (2015). Nucleocytoplasmic transport of nucleocapsid proteins of enveloped RNA viruses. Front Microbiol.

[46] Vimolmangkang S, Gasic K, Soria-Guerra R, Rosales-Mendoza S, Moreno-Fierros L, Korban SS (2012). Expression of the nucleocapsid protein of porcine reproductive and respiratory syndrome virus in soybean seed yields an immunogenic antigenic protein. Planta.

[47] Montaner S, Galiano A, Trelis M, Martin-Jaular L, Del Portillo HA, Bernal D, Marcilla A (2014). The role of extracellular vesicles in modulating the host immune response during parasitic infections. Front Immunol.

[48] Renukaradhya G, Binjawadagi B, Dwivedi V, Manickam C, Ouyang K, Torrelles J (2014). An innovative approach to induce cross-protective immunity against porcine reproductive and respiratory syndrome virus in the lungs of pigs through adjuvanted nanotechnology-based vaccination. Int J Nanomedicine.

[49] Dwivedi V, Manickam C, Binjawadagi B, Joyappa D, Renukaradhya GJ (2012). Biodegradable nanoparticle-entrapped vaccine induces cross-protective immune response against a virulent heterologous respiratory viral infection in pigs. PLoS One.

[50] Dwivedi V, Manickam C, Binjawadagi B, Renukaradhya GJ (2013). PLGA nanoparticle entrapped killed porcine reproductive and respiratory syndrome virus vaccine helps in viral clearance in pigs. Vet Microbiol.

[51] Zhu L, Song H, Zhang X, Xia X, Sun H (2014). Inhibition of porcine reproductive and respiratory syndrome virus infection by recombinant adenovirus- and/or exosome-delivered the artificial microRNAs targeting sialoadhesin and CD163 receptors. Virol J.

[52] Lener T, Gimona M, Aigner L, Börger V, Buzas E, Camussi G, Chaput N, Chatterjee D, Court FA, del Portillo HA, O’Driscoll L, Fais S, Falcon-Perez JM, Felderhoff-Mueser U, Fraile L, Song Gho Y, Görgens A, Gupta RC, Hendrix A, Hermann DM, Hill AF, Hochberg F, Horn PA, de Kleijn D, Kordelas L, Kramer BW, Krämer-Albers E-M, Laner-Plamberger S, Laitinen S, Leonardi T (2015). Applying extracellular vesicles based therapeutics in clinical trials—an ISEV position paper. J Extracell Vesicles.

