# Supplementary figures and images for: Serum-derived exosomes from non-viremic animals previously exposed to the porcine respiratory and reproductive virus contain antigenic viral proteins
Source: Vet Res. 2016 May 31;47:59. doi: 10.1186/s13567-016-0345-x (PMC4888503; doi:10.1186/s13567-016-0345-x)

Sera NV-CN-V at 2nd Ab 1/100

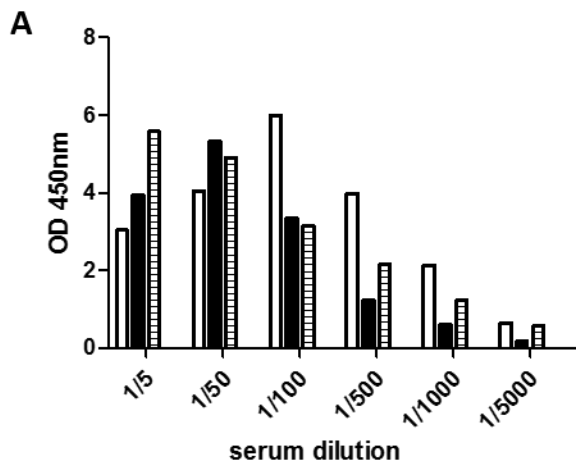

Sera NV-CN-V at 2nd Ab 1/1000

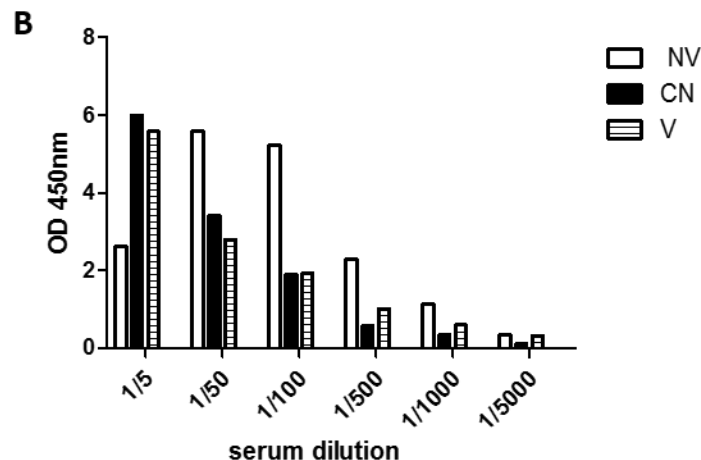

Sera NV-CN-V at 2nd Ab 1/10000

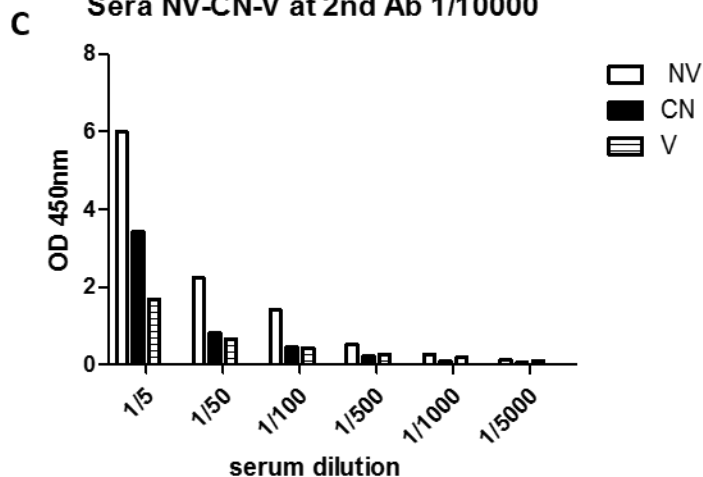

Sera NV-CN-V at 2nd Ab 1/100000

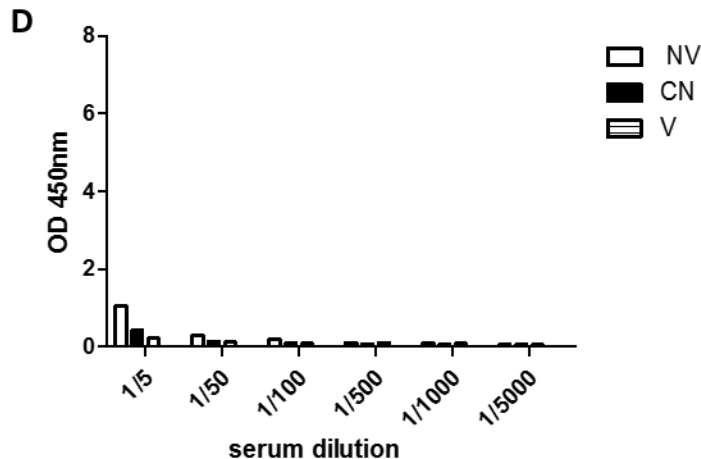

Supplement: Supplementary file 2 — 10.1186/s13567-016-0345-x ELISA chessboard plate assay for standardization of NV, CN and V porcine sera immune recognition over inactivated viral particle PRRSV vaccine (Porcilis PRRS, Intervet). Analyses of different dilutions of secondary antibody (A) 1/100, (B) 1/1000 (C) 1/10 000 and (D) 1/10 000 for each type of sera. Optical density (OD) was measured at 450nm and it is represented in the “Y” axis. “X” axis shows the dilution factor for sera samples (1/5 to 1/5000) and each bar represents a dilution for secondary antibody (1/100 to 1/100 000). The dilution used for further experiments was selected by comparison of differences between non-viremic and naïve samples and selecting the ones showing highest difference combined with highest dilution without signal saturation (1/50 for sera and 1/10 000 for secondary antibody). [file 13567_2016_345_MOESM2_ESM.pdf]

**A** Viremic sample 201406-2PS

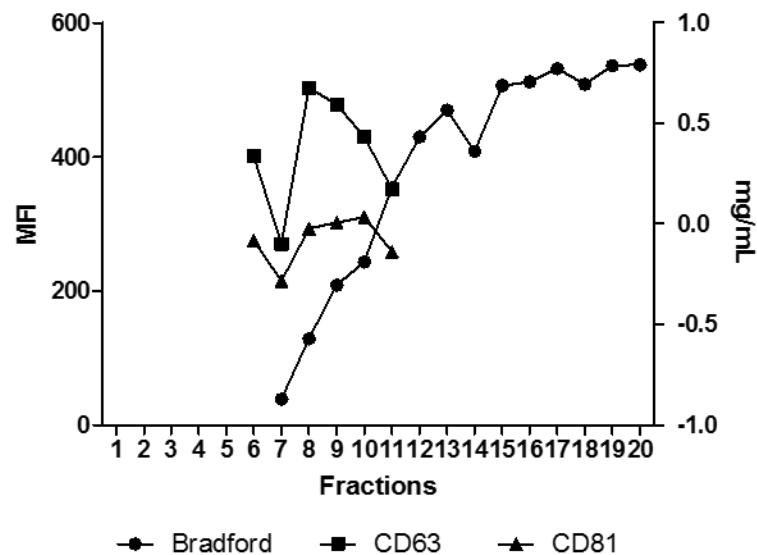

**B** Naïve sample 201406-CN2

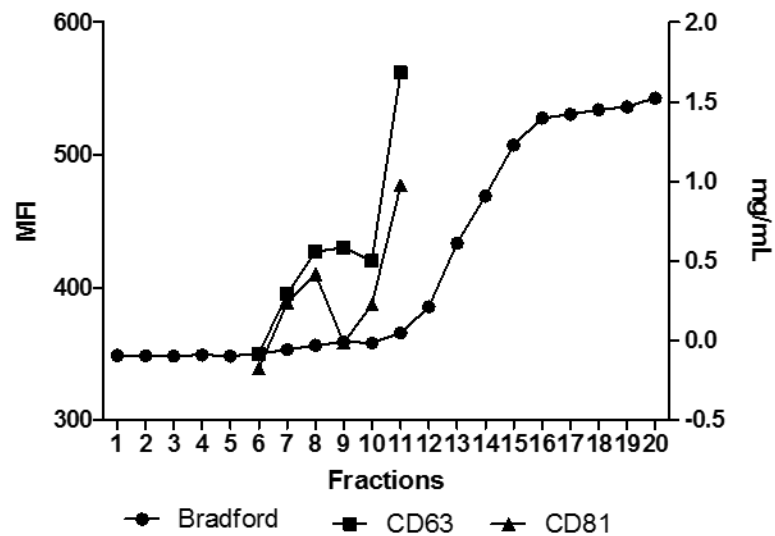

**C**

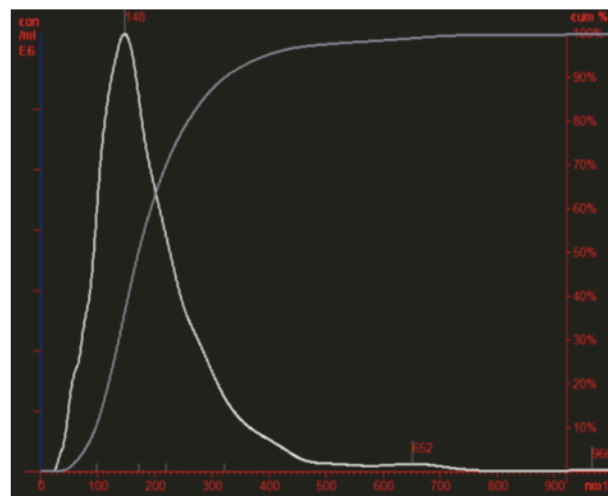

**D**

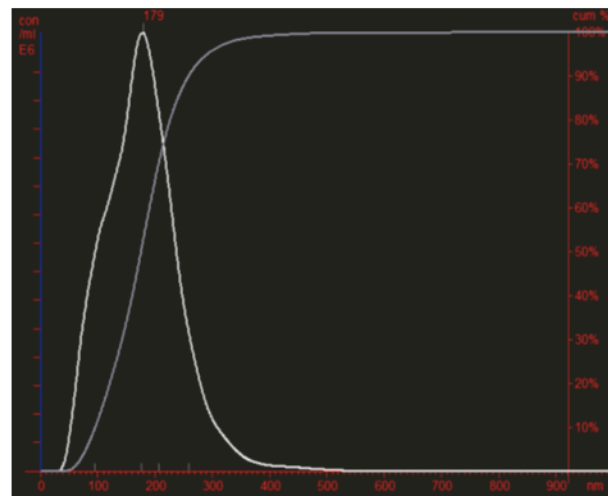

Supplement: Supplementary file 3 — 10.1186/s13567-016-0345-x Naïve and viremic protein, FACS and nanosight profile. (A) (C) Viremic sample 201406-2PS (B) (D) Naïve sample 201406-CN2. Viremic and naïve samples of sera were evaluated by the same methodology as non-viremic samples to detect any differences between groups. Protein and molecular markers profile were similar as already seen with non-viremic samples, where fractions enriched with exosomes exhibit higher MFI values for CD63 and CD81 even though there is no measurable protein detected by Bradford assay. [file 13567_2016_345_MOESM3_ESM.pdf]
