# Supplementary material for: Serum-derived exosomes from non-viremic animals previously exposed to the porcine respiratory and reproductive virus contain antigenic viral proteins
Source: Vet Res. 2016 May 31;47:59. doi: 10.1186/s13567-016-0345-x (PMC4888503; doi:10.1186/s13567-016-0345-x)
Supplement: Supplementary file 5 — 10.1186/s13567-016-0345-x Venn diagram of farm animal exosomal proteins and coincidence table comparing Sus scrofa and Bos taurus. Exosome proteins from different farm animal species obtained from Vesiclepedia were used to compare with our Sus scrofa exosomal proteins. Similarities among species in exosomal proteins are represented in the venn diagram. [file 13567_2016_345_MOESM5_ESM.pdf]

	Sus scrofa	Bos taurus	Equus caballus	Gallus gallus	Rattus norvegicus
Sus scrofa					
Bos taurus	48 (2.9%)				
Equus caballus	5 (2.5%)	18 (1.1%)			
Gallus gallus	6 (2.8%)	35 (2.2%)	1 (0.8%)		
Rattus norvegicus	3 (0.2%)	6 (0.2%)	1 (0.1%)	0 (0.0%)	
